# Longitudinal Study Detects the Co-Carriage of ESBL and *mcr*-*1* and -*4* Genes in *Escherichia coli* Strains in a Portuguese Farrow-to-Finish Swine Herd

**DOI:** 10.3390/ani12172209

**Published:** 2022-08-27

**Authors:** Tiago Lima, Laura Fernandes, Marta Matias, Ana Mateus, Eduarda Silveira, Sara Domingues, Constança Pomba, Gabriela Jorge Da Silva

**Affiliations:** 1 Faculty of Pharmacy, University of Coimbra, 3000-458 Coimbra, Portugal; 2 Center for Neuroscience and Cell Biology, University of Coimbra, 3004-517 Coimbra, Portugal; 3 Centre for Interdisciplinary Research in Animal Health, Laboratory of Antibiotic Resistance, CIISA, Faculty of Veterinary Medicine, University of Lisbon, 1300-477 Lisboa, Portugal; 4 Department of Veterinary Medicine, Veterinary School Vasco Da Gama, 3020-210 Coimbra, Portugal

**Keywords:** antimicrobial resistance, swine, colistin, public health, livestock

## Abstract

**Simple Summary:**

The difficulty of treating bacterial infections has become a worldwide health concern due to the evolution of bacterial antibiotic resistance. Antibiotics are routinely used to prevent and treat infections in food-producing animals, especially in young animals. This procedure may lead to resistant bacteria in the animal gut that may indirectly cause a broader public health issue. Therefore, we conducted a study in a pig farrow-to-finish operation during five months to understand how the administration of antibiotics in sows and piglets impact the emergence of antibiotic resistance in *Escherichia coli* and its maintenance in the gut until the slaughter weight is reached. Our results showed that sows and their piglets carried antibiotic resistant *E. coli* throughout their life cycle that can be associated to farm administration of antibiotics. These antibiotics are critical in human medicine, which includes the antibiotic colistin used as a last-resort therapy in some multidrug resistant bacteria in human infections. In addition, these bacteria demonstrated the presence of virulence genes that support pathogenesis. Overall, this study highlights the need to reduce antibiotic use in animal husbandry or to find alternatives to antibiotics in food-animal production to reduce the emergence and potential spread of antibiotic–resistant pathogens.

**Abstract:**

Cephalosporins and polymyxins are employed in antimicrobial protocols to control and treat neonatal infections and post-weaning diarrhoea in swine operations. We conducted a longitudinal study to evaluate the colonization and transmission of antibiotic–resistant *Escherichia coli* in sows and their piglets in a farrow-to-finish operation, focusing on characterization of Extended Spectrum Beta-Lactamase (ESBL) and *mcr* genes, virulence traits and genetic relatedness. A total of 293 *E. coli* isolates were obtained from faecal samples collected in five time points. At birth *bla*_CTX-M-1group_ cluster was detected in *E. coli* isolates from 9 sows and 49 piglets (73.41%), while in the following four’ piglets sampling moments it was detected in 91.8%, 57.6%, 71.4% and 97.4%. The gene *mcr*-*1* was detected in *E. coli* from one sow and from three piglets from different litters at birth and increased in the first weeks of piglet life (68.85%, 100%, 90% and 8.1%). A new *mcr*-*4* allele, *mcr*-*4.7*, was identified in 3.28%, 28.57%, 7.5% of *E. coli* isolates. Most *mcr*-positive *E. coli* isolates (96,7%) carried *bla*_CTX-M-1Group_ genes and 93,33% carried both *mcr*-*4* and *mcr*-*1*. CTX-M-1 and CTX-M-32 were the most predominant ESBLs. Plasmids belonged to IncI1, IncF and IncN groups. Most isolates belong to phylogenetic group B1; PAI IV_536_ marker was detected in nine isolates. The strains were kept in the different stages of the piglets’ life. The use of ceftiofur and colistin may explain the high prevalence and co-selection of *bla*_CTX-M-1Group_ and *mcr*-*1* and/or -*4* genes, contributing to the maintenance of resistant and virulent isolates throughout the pig life cycle that may reach the food chain.

## 1. Introduction

The ever-increasing emergence and dissemination of antimicrobial resistance is a huge concern in clinical, veterinary and environmental settings worldwide. Due to global demographic growth and consequent increasing demand for livestock products, livestock production has become intensive, with significant improvements in breeding, nutrition and animal health [1,2].

In veterinary medicine and intensive farming production, antibiotics are largely used not only with prophylactic, metaphylactic and therapeutic purposes but are also used in subtherapeutic doses as growth promotants, despite tight restrictions in some countries associated with this practice. In the particular case of intensive pig husbandry systems, for many years, antimicrobials, such as polymyxins, β-lactams and aminoglycosides, have been used prophylactically during the nursery period to control Gram-negative infections in piglets [3]. Colistin and third-generation cephalosporins, which are classified by the World Health Organization as “Highest Priority Critically Important Antimicrobials” for human medicine [4] are now not allowed to be used as prophylactic agents in animals. Third-generation cephalosporins such as ceftiofur are a part of the beta-lactam antibiotic class and are some of the most widely used antibiotics approved for systemic treatment of bacterial infections in intensive animal production [5]. The veterinary administration of extended-spectrum cephalosporins for extended-spectrum β-lactamase (ESBL)-producing *Enterobacterales* in animals may increase zoonotic transmission of resistant bacteria carrying plasmid-mediated ESBL resistance genes [6], therefore acting as potential reservoirs of clinically important antibiotic-resistance genes through the food-producing animals’ gut microbiota [5].

Another antibiotic that has been widely used in animal husbandry due to its efficiency and low cost is colistin, administered mainly during the nursery period [3,7]. Colistin use in the human hospital setting has also become one of the last resorts in the treatment of bacterial infections caused by multidrug-resistant (MDR) bacteria, in particular of carbapenemase-producing *Enterobacterales* [8]. Until 2015, prevalence of colistin resistance was considered low and all known resistance mechanisms were chromosomally encoded [7]. However, a plasmid-mediated colistin resistance gene (*mcr*-*1* gene) was identified for the first time as part of a conjugative plasmid in *Escherichia coli* isolates of animal origin from China [9]. Since then, plasmid-borne *mcr* alleles have been reported in numerous studies, mostly detected in *E. coli* of animal origin [10].

Faecal carriage of ESBL and *mcr* genes has been widely identified in both the human clinical setting and animal reservoirs [10,11], and while resistance levels to third generation cephalosporins and colistin remains generally low in pork meat produced in the European Union (EU) [12], the food chain may significantly increase the dissemination and acquisition of third generation cephalosporin and colistin resistance worldwide [13]. In 2020, the main meat produced in the EU was pork, with 23 million tonnes, nearly doubling the quantity of the second and third top meat protein sources produced (13.6 million tonnes of poultry and 6.8 million tonnes of beef, respectively) [14]. These data highlight the need for food safety measures and surveillance in farrow-to-finish swine herds due to the major role of pork in human food and also the need to understand if antibiotic use in pigs allows the selection of resistant strains to be carried from birth through the production cycle until slaughter and ultimately enter the food chain. We performed a longitudinal study during 5 months to assess the prevalence and transmission of ESBL and *mcr*-producing *E. coli* in the sows and their piglets from farrow-to-finish in Portuguese intensive farm production, characterizing their antimicrobial resistance, virulence features and genetic relatedness.

## 2. Materials and Methods

### 2.1. Selection of Herds and Sampling Scheme

This study was conducted on an industrial swine farm located in Portugal in the Alentejo region, where ceftiofur was routinely used. A total of 10 sows with newly born litters were randomly selected, and 7 new-born healthy piglets from each corresponding litter were ear-tagged with a code for litter (letter A–J) and piglet (number 1–7) identification (*n* = 79). One of these newborn piglets died after being tagged. A single dose of ceftiofur (Naxcel^®^ 20 mg/piglet IM) was prophylactically administered to all new-borns to prevent neonatal infections, namely *Streptococcus suis* infections, navel infections, arthritis and colibacillosis diarrhoea. Colistin was also administered as part of the ongoing antimicrobial protocol for post-weaning diarrhoea due to *E. coli*.

Faecal samples were collected from each sow when giving birth. Piglet samples were collected with sterile swabs at 5 time points over 5 months between November 2011 and April 2012: (i) at birth (before ceftiofur administration); (ii) after weaning; (iii) at the nursery unit; (iv) at the finishing unit; and (v) before leaving the finishing unit (one day before transportation to the abattoir).

### 2.2. Isolation and Identification of Escherichia coli

Each faecal sample was enriched for 18 h at 37 °C in buffered peptone water. Subsequently, 100 µL of the bacterial suspension were inoculated onto MacConkey agar supplemented with 1.5 µg/mL of cefotaxime and incubated aerobically overnight at 37 °C. *E. coli* isolates were presumptively identified by phenotypic features, namely colony morphology and lactose-fermenting ability, and their identity was confirmed by screening for *gadA* gene by PCR, as previously described [15].

### 2.3. Antimicrobial Susceptibility Testing

The antimicrobial susceptibility testing was performed by disk diffusion method according to the guidelines of the Performance Standards for Antimicrobial Susceptibility Testing, using the following antimicrobial-containing disks: 10 µg amoxicillin/clavulanic acid (Amc), 10 µg amoxicillin (Aml), 30 µg cefotaxime (Ctx), 30 µg ceftazidime (Caz), 30 µg cefoxitin (Fox) and 30 µg ceftiofur (Xnl) according to the Clinical Laboratory Standards Institute guidelines M31-A3 [16], M100-S22 [17] and recommendations of the French Microbiology Society for veterinary antimicrobial susceptibility testing [18]. Phenotypic detection of ESBL was carried out by the double-disk synergy test [17]. *E. coli* ATCC 25,922 (American Type Culture Collection) was used as a quality control strain.

For extended phenotypic and genotypic characterization, 60 isolates obtained from 10 piglets, from each sow at each sampling moment were selected. Susceptibility to other antibiotics was also tested by disk diffusion method using disks containing 30 µg nalidixic acid (Na), 5 µg ciprofloxacin (Cip), 5 µg enrofloxacin (Enr), 25 µg sulfamethoxazole/trimethoprim (Sxt), 30 µg chloramphenicol (C), 30 µg florfenicol (Ffc), 200 µg fosfomycin (Fos), 300 µg nitrofurantoine (F), 10 µg gentamicin (Cn), 30 µg amikacin (Ak), 10 µg imipenem (Imp) and 10 µg meropenem (Mem) disks. The results were interpreted according to the abovementioned guidelines with the exception of florfenicol susceptibility, which was interpreted according to Keyes et al. [19].

### 2.4. Detection of Antimicrobial Resistance Genes

Bacterial isolates that grew in cefotaxime supplemented MacConkey agar were screened for *bla*_CTX-M_ type-encoding genes by PCR as previously described [20]. The *bla*_CTX-M_ negative isolates were screened for other common ESBL genes, namely *bla*_TEM_, *bla*_OXA_ and *bla*_SHV_ type genes by a multiplex PCR, according to Pomba et al. [21]. Identification of the specific *bla*_CTX-M_ gene was carried out by DNA sequencing of the amplicon generated with primers designed for the *bla*_CTXM-1-group_ [21].

The detection of *mcr*-*1* to -*9* genes was performed in all isolates by three multiplex PCRs: one for screening *mcr*-*1* and *mcr*-*3;* the second for *mcr*-*2*, *mcr*-*4* and *mcr*-*5* and the third for *mcr*-*6* to *mcr*-*9*, using the primers described by Rebelo et al. [22] and Borowiak et al. [23], with some PCR methodology modifications. The *mcr*-*10* was screened by simplex PCR using the in-house designed primers: *mcr*-*10*_fw [5′-ATTCCGTTTGTGCTGGTTGC-3′] and *mcr*-*10*_rv [5′-AACATACAGGGCACCGAGAC-3′] and the following conditions: a cycle of denaturation at 95 °C for 60 s, followed by 30 cycles of denaturation at 95 °C for 30 s, annealing at 58 °C for 30 s and elongation at 72 °C for 60 s, and a final cycle of elongation at 72 °C for 10 min. The expected *mcr*-10 amplicon size was 707 base pairs. On *mcr*-*4*-positive isolates, we performed another *mcr*-*4* amplification using the external primers designed by Carattoli et al. [24] (Table S1). All the amplicons of *mcr* genes were sequenced on both strands (Stabvida, Portugal), and sequences were compared with those included in the GenBank database.

### 2.5. Genetic Relatedness and Virulence Markers Detection

Genetic relatedness was evaluated in the isolates (*n* = 59) from the 10 piglets selected for extended characterization by pulsed-field gel electrophoresis (PFGE) analysis, following the PulseNet Protocol [25]. Total DNA from *E. coli* isolates was digested using the *XbaI* restriction enzyme. The generated fragments were separated by PFGE using a CHEF-DR III System (Bio-Rad, San Diego, CA, USA, EUA). PFGE pattern analysis was performed with BioNumerics software v 4.61 (Applied Math, Ghent, Belgique) using Dice’s coefficient and the unweighted pair group method with arithmetic mean (UPGMA dendrogram type). Pulsed-field type clusters and subtypes were assessed according to the settings recommended by Carriço et al. with a position tolerance of 1.7% for type classification and 2.5% for subtype, based on a similarity cut-off of ≥80% [26]

*E. coli* isolates were also classified into one of the four main phylogenetic groups A, B1, B2 and D, following the PCR-based technique described previously [27].

Plasmid incompatibility groups were identified among isolates by PCR-based replicon typing, using 18 pairs of primers [28].

A total of 8 pathogenicity island markers were screened by multiplex PCR [29,30]; with a few modifications. Briefly, PCR assays were split in 3 separate multiplex assays: multiplex A for PAI III_536_, PAI IV_536_ and PAI II_CFT073_; multiplex B1 for PAI II_J96_ and PAI I_536_; and multiplex B2 for PAI II_536_, PAI I_CFT073_ and PAI I_J96_.

## 3. Results

### 3.1. Occurrence of bla_CTX-M_ and mcr Type Genes and Other Resistance Genes in E. coli

**Figure 1:**
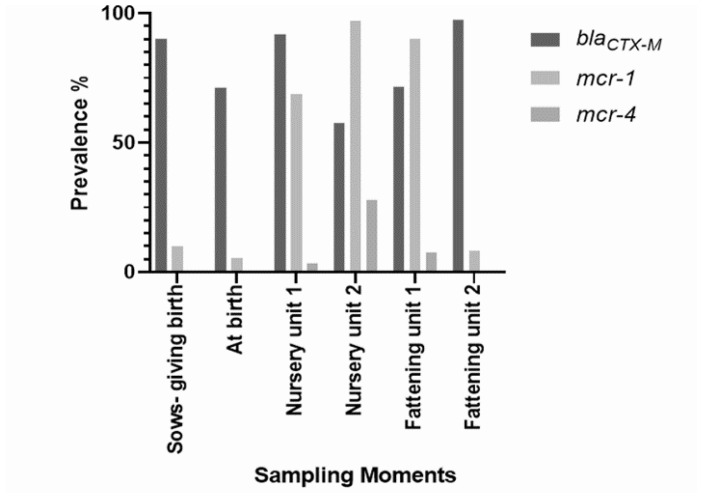
Prevalence of *bla*_CTX-M_ and *mcr*-producing *E. coli* throughout the piglets’ life. The first sampling moment refers to the sows, when they give birth (*n* = 10). The following five sampling moments refer to the piglets from farrow-to-finish. At each sampling moment, *E. coli* isolates were selected by phenotypic features on cefotaxime supplemented MacConkey agar, and their identity was confirmed by *gadA* PCR, which led to obtaining f a different number of isolates over the time of the study.

From all sampling moments over five months of study, a total of 293 *E. coli* isolates grew in cefotaxime supplemented MacConkey agar and were screened for *bla*_CTX-M-_ type genes. At birth, *bla*_CTX-M-1Group_ type genes were detected in 90% of sows (*n* = 9) and 71% of piglets (*n* = 49). Additionally, 10% of sows (*n* = 1) and 5.36% of piglets (*n* = 3) from different litters also carried the *mcr*-*1* gene. In the following four sampling moments of piglets, *bla*_CTX-M_ type genes were detected in 91.8% (*n* = 56) after weaning, 57.62% (*n* = 34) at the nursery unit, 71.42% (*n* = 40) at the finishing unit and 97.36% (*n* = 37) before leaving the finishing unit. The *mcr* type-encoding genes were screened in 234 of 317 isolates. Only *mcr*-*1* and *mcr*-*4* were identified. The *mcr*-*1* gene was detected in 68.85% (*n* = 42), 97.22% (*n* = 35), 90% (*n* = 36) and 8.10% (*n* = 3) of isolates obtained after weaning, at the nursery, at the finishing unit and before leaving the finishing unit, respectively. The *mcr*-*4* gene was identified in 2 (3.3%), 10 (27.8%) and 3 (7.5%) of the isolates collected after weaning, at the nursery unit and at the finishing unit, respectively. Nucleotide sequencing of the *mcr*-*4* amplicons performed on both DNA strands revealed a new allele of the *mcr*-*4* gene. Compared to the prototype *mcr*-*4.1* gene (GenBank accession no. MF543359.1) [24], this new allele contains three missense mutations at positions 706 (G706T), 992 (A992G) and 1453 (G1453A) resulting in amino acid transitions Val236Phe, Gln331Arg and Val485Ile, respectively, as shown in Table S2. The sequence of this new allele, named *mcr*-*4.7*, is released at the NCBI GenBank under accession number ON586856.1. Figure 1 illustrates the prevalence of the *bla*_CTX-M_ type, the *mcr*-*1* and the *mcr*-*4* genes at each sampling moment.

Remarkably, most *mcr*-positive isolates (96.7%) also carried *bla*_CTX-M_ type genes and 93.3% of *mcr*-*4* producing isolates also harboured the *mcr*-*1* gene. Furthermore, at birth, three *bla*_CTX-M_ positive isolates also showed cefoxitin resistance, and in one cefotaxime-resistant *bla*_CTX-M_ negative isolate, the *bla*_TEM_ type gene was detected instead.

### 3.2. Extended Phenotypic and Genotypic Characterization

#### 3.2.1. Antimicrobial Resistance

A total of 60 isolates from 10 pigs were selected to test antibiotic susceptibility to drugs other than β-lactams. Overall, all isolates were susceptible to amoxicillin/clavulanic acid combination, fosfomycin, amikacin and to carbapenems; in contrast, they were resistant to amoxicillin, cefotaxime and ceftiofur. A total of 22 different resistance patterns were found in the farm during the period of study. The most frequently detected pattern (17 isolates) comprised resistance to amoxicillin, cefotaxime, and ceftiofur, followed by the amoxicillin, cefotaxime, ceftazidime, ceftiofur, sulfamethoxazole/trimethoprim, chloramphenicol, florfenicol and gentamicin resistance pattern, detected in 7 isolates. Table 1 shows the number of different resistance patterns, as well the most prevalent antimicrobial resistance profile found in each of the six sampling moments.

#### 3.2.2. Identification of bla_CTX-M_ Genes and Plasmid Incompatibility Groups

Nucleotide sequencing of 60 *bla*_CTX-M_ type genes revealed that 32 *E. coli* isolates carried *bla*_CTX-M-1_ (53.33%), and 28 carried *bla*_CTX-M-32_ (46.67%) (Table 1). Additionally, by using PCR-based replicon typing, plasmids were assigned to IncI1, IncF, and IncN groups, the latter being the least frequent. In *bla*_CTX-M-32_-carrying isolates, IncF plasmids were detected in 19, followed by IncI1 plasmids (*n* = 8) and IncN plasmids (*n* = 1), while in *bla*_CTX-M-1_-carrying isolates IncI1 plasmids (*n* = 21) was more prevalent, followed by IncF (*n* = 8) and IncN (*n* = 3) plasmids.

Otherwise, IncF (*n* = 15) and IncI1 (*n* = 12) plasmids were identified in *mcr*-*1*-carrying isolates, while IncF (*n* = 2) and IncI1 (*n* = 1) plasmids were detected in the new allele *mcr*-*4*-harbouring isolates.

#### 3.2.3. Pulsed-Field Gel Electrophoresis (PFGE)

The clonal relationship of 59 *E. coli* isolates from piglets over the five sampling moments was assessed by PFGE. Different genetic profiles were observed and 16 genetic clusters were found. The dendrogram analysis showed that genetically closely related isolates were maintained through the different stages of piglets’ life (Figure S1).

#### 3.2.4. Pathogenicity Island Markers and Phylogenetic Analysis

Strain virulence was inferred by detection of pathogenicity islands and phylogeny.

PAI markers were detected in nine isolates (15.0%) as shown in Table 1. Notably, one isolate carried both PAI IV_536_ and PAI I_CFT073_ markers, while the other eight isolates only carried PAI IV_536_ marker. The other PAI markers targeted by this study were not detected in any of the isolates.

These *E. coli* isolates were allocated into four phylogenetic groups. The B1 phylogenetic group was the most prevalent (*n* = 45; 75.0%), followed by group A (*n* = 10; 16.67%). The phylogenetic group B2 and D was only found in two (3.33%) and one (1.67%) isolates, respectively.

## 4. Discussion

This study describes the characterization of *E. coli* strains from pigs in an intensive farm production setting from birth to finishing, using both phenotypic and genotypic methods to characterize and study the dynamics of resistant *E. coli* persistence and dissemination at animal level in livestock production.

A high prevalence of *bla*_CTX-M_ genes was found in *E. coli* throughout the piglet’s life cycle in the Portuguese farm included in this study. Similar data were reported from diverse intensive farming production from diverse European countries where, likewise, ceftiofur was used as prophylactic treatment [31,32,33,34]. In this study, *bla*_CTX-M-1_ was the most prevalent gene, followed by *bla*_CTX-M-32_, which is in agreement with other studies conducted in Europe [35,36].

The colistin-resistant *mcr*-genes screening revealed a high prevalence of the *mcr*-*1* gene. In fact, the *mcr*-*1* gene has been widely reported in *E. coli* strains from food-producing animals, mainly from the pig reservoir [5,22,24,37,38]. Although *mcr*-*4* was firstly described in 2017 [24] in pig samples, in our study *mcr*-*4* was detected mainly in *mcr*-*1*-producing *E. coli* strains isolated in 2011. To our knowledge, this is the first time the *mcr*-*4* gene is reported in Portugal. Moreover, the sequencing of the gene revealed a new *mcr*-*4* allele, which contains three point mutations when compared with the *mcr*-*4.1* allele [24]. Some point mutations were previously described; however, this is the first report of the mutation at position 1453, which results in the change of amino acid 485 due to the substitution of a valine for an isoleucine [39,40].

There are only a few reports of co-occurrence of *mcr*-*1* and *mcr*-*4*, but they are all from swine isolates from Spain [22,37,41,42]. Thus, this is the first report of *E. coli* isolates harbouring *bla*_CTX-M-32_ or *bla*_CTX-M-1_, *mcr*-*1* and *mcr*-*4* recovered from Portuguese piglets.

In this study, a high prevalence of *bla*_CTX-M_ genes was found both in the newborn piglets before ceftiofur administration and in sows when they give birth. This finding suggests that vertical transmission of the resistant bacteria from sows to the offspring has occurred. Transmission of bacteria during perinatal period remains unclear. However, some studies showed that primary colonisation of newborn piglets’ gut is influenced by the mother and the environmental microbiota in the farrowing unit [43,44]. The maintenance of resistant *E. coli* during all stages of the piglets lives and the increased levels of *bla*_CTX__-M_ at the nursery unit 1 may be due to the prophylactic administration of ceftiofur to all piglets immediately after birth, highlighting the need of alternative practices to prevent young animal infections. Similarly, high levels of prevalence of *mcr* genes were observed during the after weaning, at the nursery and at the finish units. In this farm, colistin was administered for post weaning diarrhoea that may affect piglets during the after weaning period, associated with intestinal dysbiosis and proliferation of enterotoxigenic *E. coli*. Therefore, colistin pressure may be responsible for the emergence of *mcr* genes, since in the first sampling moments and in the sows, these genes were present at low rates. The levels of *mcr* genes tended to be less frequent as time after treatment progresses. Thus in the beginning of the fattening period, a small reduction in the prevalence of the *mcr* genes was observed and a sharp decrease in this prevalence was found before leaving the finish unit. Finally, it is important to note that the detection of these resistance genes was performed in the isolates obtained after selective culturing with cefotaxime and not with colistin. Therefore, the prevalence of *mcr* genes in this study may be underestimated.

The use of ceftiofur was not only associated with amoxicillin and cefotaxime resistance but also with structurally unrelated antimicrobials such as chloramphenicol [45]. In this study, resistance to chloramphenicol was observed in 35 isolates of piglets (58.33%), despite no phenicols being used in the early stages of the piglets’ lives. Indeed, in previously reported studies, chloramphenicol resistance genes have often been found on the same ESBL encoding plasmids [45,46,47,48,49].

The *bla*_CTX-M-1_ and *bla*_CTX-M-32_ detected in this study were likely located on IncI1, IncF and IncN plasmids, which means that one ESBL-type gene is possibly mobilized by different incompatibility plasmids,, and at the same time one incompatibility plasmid group can possibly carry different ESBL-type genes. The association of *bla*_CTX-M_ type genes and the conjugative plasmids of incompatibility groups A/C, F, HI, I1, L and N has been observed. In fact, *bla*_CTX-M-1_-carrying Inc N, Inc F and IncI1 plasmids are strongly associated with *Enterobacterales* isolated from animal sources and these plasmids play a major role in dissemination of these antimicrobial resistance genes among the bacteria population [50]. Beyond the resistance transfer, we aimed to understand whether the same strains were maintained along the pig life cycle. Indeed, the same *E. coli* clones were found in different sampling moments, from the sows to the finishing unit, showing the maintenance of the resistant and virulent isolates throughout a pig’s life cycle and demonstrating that piglets not only acquire *E. coli* clones from their mothers but also act as active carriers of possibly plasmid-borne *bla*_CTX-M_ type and *mcr E. coli* strains.

The majority of *E. coli* isolates were allocated into the B1 phylogenetic group, which includes intestinal pathogenic strains with increased ability to persist in the environment [51]. The B1 phylogenetic group is usually considered less virulent than strains from the phylogenetic groups B2 and D [49]. However, in this study nine *E. coli* isolates carry one or more PAI markers. PAI IV_536_ encoding for the yersiniabactin iron-uptake system was the most frequently detected, which is in concordance with previous studies reporting PAI IV_536_ as the most ubiquitous in *Enterobacterales* [29,52]. Often it is detected together with PAI I_CFT07_ [50]. However, PAI IV_536_ was mostly detected alone in this study, mostly in B1 phylogenetic group. This high frequency of PAI IV_536_ in commensal isolates has led to the suggestion that PAI IV_536_ may be a fitness island rather than a pathogenicity one [29]. From our point of view, the carriage of siderophore, a virulence factor, will increase the fitness of the bacteria, and it will contribute to the maintenance of potential pathogenic strains along the productive cycle of pigs that, ultimately, may enter in food chain.

## 5. Conclusions

Overall, a high proportion of sows and their piglets were colonized by ESBL/*mcr*-producing *E. coli*. The results of this longitudinal study showed that the use of antibiotics in intensive animal production in the early stages of life, namely ceftiofur and colistin, exert a high selective pressure in pig gut microbiota, and as such contributing to the high prevalence and co-selection of *bla*_CTX-M-*1Group*_ and *mcr*-*1* and/or -*4* genes in *E. coli* strains and promoting the maintenance of resistant and virulent strains throughout the pig life cycle, potentially reaching humans through the food chain. This highlights the importance of antimicrobial stewardship during all stages of a production animal’s life as a possible tool to achieve a reduction of the burden of multidrug-resistant and virulent isolates.

## Figures and Tables

**Table 1:** Characteristics of *bla*_CTX-M_ producing *E. coli* isolates recovered from piglets from a Portuguese farms over 5 sampling moments.

Sampling Moment	*n*	*bla*_CTX-M_ Gene		*mcr* Gene		Phylogenetic Group					PAI Marker Profile	Number of Resistance Patterns	Antimicrobial Resistance Profile ^a^
		*bla*_CTX-M-1_	*bla*_CTX-M-32_	*mcr*-*1*	*mcr*-*4*	A	B1	B2	D	NT			
At birth	10	5	5	0	0	4	5	0	0	1	IV_536_ (2)	7	AmlCtxXnlSxt (3)
Nursery 1	10	5	5	7	0	2	5	2	0	1	-	8	AmlCtxXnl (2) AmlCtxXnlNaEnrCFfcCn (2)
Nursery 2	10	3	7	10	2	0	10	0	0	0	IV_536_ (3)	8	AmlCtxXnlNaCCn (2) AmlCtxXnlNaCipEnrSxtCCn (2)
Fattening unit 1	10	1	9	9	1	0	10	0	0	0	IV_536_ (3)	4	AmlCtxXnlCazSxtCFfcCn (6)
Fattening unit 2	10	8	2	1	0	3	7	0	0	0	-	3	AmlCtxXnl (7)

Abbreviations: Aml, amoxicilin; Ctx, cefotaxime; Xnl, Ceftiofur; Sxt, Sulfamethoxazole/trimethoprim; Ffc, Florfenicol; Na, Nalidixic acid; Enr, Enrofloxacin; C, Chloramphenicol; Cn, Gentamicin; Cip, Ciprofloxacin; Caz, Ceftazidime; Fox, Cefoxitin; F, Nitrofurantoine; ^a^ most frequent antimicrobial resistance profile; *n* = number of positive *bla_CTM_*_-*M*_ producing *E. coli* isolates (*n*_total_ = 60); NT = not typeable.

## Data Availability

Not applicable.

## Supplementary Materials

The following are available online at https://www.mdpi.com/article/10.3390/ani12172209/s1, Figure S1: Dendrogram of clonal relationship of *E. coli* isolates from piglets over the five sampling moments. Table S1: Primers used in this study for detection of *gadA/B, bla*_CTX-M_, *bla*_TEM_, *bla*_SHV_, *bla*_OXA-1_, *ampC* and *mcr*-*1* to -*10* genes. Table S2: Nucleotide/amino acid changes in *mcr*-*4*/MCR-4 alleles.
