# Supplementary material for: Longitudinal Study Detects the Co-Carriage of ESBL and mcr-1 and -4 Genes in Escherichia coli Strains in a Portuguese Farrow-to-Finish Swine Herd
Source: Animals (Basel). 2022 Aug 27;12(17):2209. doi: 10.3390/ani12172209 (PMC9454694; doi:10.3390/ani12172209)
Supplement: Supplementary file 1 [file animals-12-02209-s001.zip › animals-1869381-supplementary.pdf]

Table S1. Primers used in this study for detection of *gadA/B*, *bla*CTX-M, *bla*TEM, *bla*SHV, *bla*OXA-1, *ampC* and *mcr-1* to *-10* genes

**Primer name**	**Sequence (5′-3′)**	**Target gene**	**Amplicon size (bp)**	**Reference**
gadA/B_fw<br>gadA/B_rv	ACCTGCGTTGCGTAAATA<br>GGGCGGGAGAAGTTGATG	*gadA* and *gadB*	670	[15]
CTX-M_fw<br>CTX-M_rv	TTTGCGATGTGCAGTACCAGTAA<br>CGATATCGTTGGTGGTGCCATA	*bla*CTX-M genes	544	[20]
TEM_fw<br>TEM_rv	TTCCTGTTTTTGCTCACCCA<br>TACGATACGGGAGGGCTTAC	*bla*TEM	716	[21]
SHV_fw<br>SHV_rv	TCAGCGAAAAACACCTTG<br>TCCCGCAGATAAATCACCA	*bla*SHV	471	
OXA-1_fw<br>OXA-1_rv	TATCTACAGCAGCGCCAGTG<br>CGCATCAAATGCCATAAGTG	*bla*OXA-1	199	
ampC_fw<br>ampC_rv	CCCCGCTTATAGAGCAACAA<br>TCAATGGTCGACTTCACACC	*ampC*	634	
mcr1_320bp_fw<br>mcr1_320bp_rev	AGTCCGTTTGTTCTTGTGGC<br>AGATCCTTGGTCTCGGCTTG	*mcr-1*	320	[22]
mcr2_700bp_fw<br>mcr2_700bp_rev	CAAGTGTGTTGGTCGCAGTT<br>TCTAGCCCGACAAGCATACC	*mcr-2*	715	
mcr3_900bp_fw<br>mcr3_900bp_rev	AAATAAAAATTGTTCCGCTTATG<br>AATGGAGATCCCCGTTTTT	*mcr-3*	929	
mcr4_1100bp_fw<br>mcr4_1100bp_rev	TCACTTTCATCACTGCGTTG<br>TTGGTCCATGACTACCAATG	*mcr-4*	1,116	
MCR5_fw<br>MCR5_rev	ATGCGGTTGTCTGCATTTATC<br>TCATTGTGGTTGTCCTTTTCTG	*mcr-5*	1,644	
mcr-6_mp_fw<br>mcr-6_mp_rev	AGCTATGTCAATCCCGTGAT<br>ATTGGCTAGGTTGTCAATC	*mcr-6*	252	[23]
mcr-7_mp_fw<br>mcr-7_mp_rev	GCCCTTCTTTTCGTTGTT<br>GGTTGGTCTCTTTCTCGT	*mcr-7*	551	
mcr-8_mp_fw<br>mcr-8_mp_rev	TCAACAATTCTACAAAGCGTG<br>AATGCTGCGCGAATGAAG	*mcr-8*	856	
mcr-9_mp_fw<br>mcr-9_mp_rev	TTCCCTTTGTTCTGGTTG<br>GCAGGTAATAAGTCGGTC	*mcr-9*	1011	
mcr-10_fw<br>mcr-10_rev	ATTCCGTTTGTGCTGGTTGC<br>AACATACAGGGCACCGAGAC	*mcr-10*	707	This study
Mcr-4 ext FW<br>Mcr-4 ext RV	ATCTGTTAAGTTTGTTGGTGAC<br>TGAGAGCTAAATGTAACAATAGA	complete *mcr-4*	1,820	[24]

Table S2. Nucleotide/amino acid changes in *mcr-4*/MCR-4 alleles.

Allele	Nucleotide Mutation						Amino acid changes	Accession no.
	**329**	**536**	**613**	**706**	**992**	**1453**		
4.1	C	T	C	G	A	G	–	MF543359
4.2	C	T	C	G	**G**	G	Gln331Arg	MG822663
4.3	C	**G**	C	**T**	A	G	Val179Gly, Val236Phe	MG026621
4.4	C	T	**A**	G	**G**	G	His205Asn, Gln331Arg	MG822665
4.5	**T**	T	C	G	**G**	G	Pro110Leu, Gln331Arg	MG822664
4.6	C	T	C	**T**	A	G	Val236Phe	MH423812
4.7	C	T	C	**T**	**G**	**A**	Val236Phe, Gln331Arg, Val485Ile	ON586856

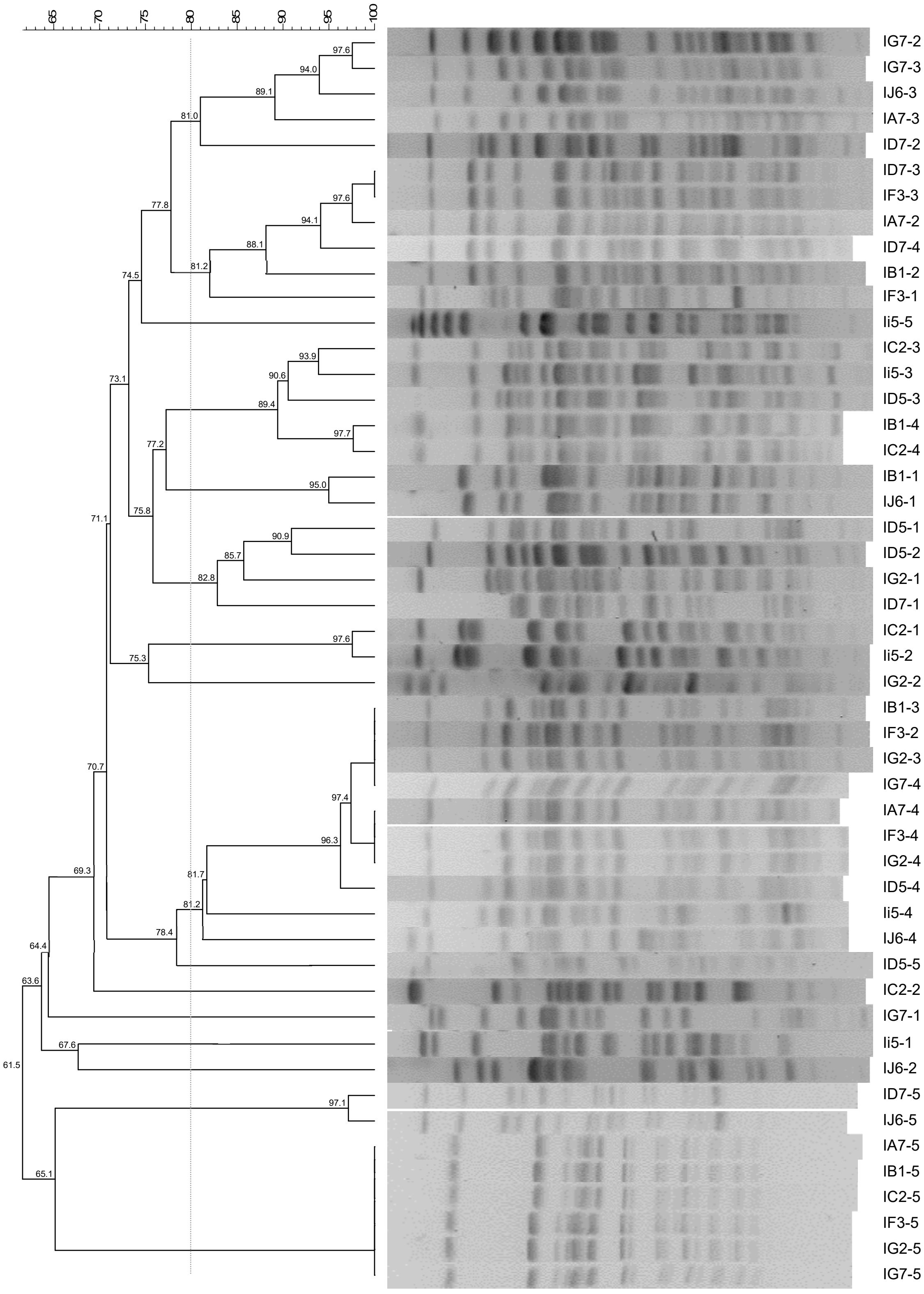

Figure S1. Dendrogram of clonal relationship of *E. coli* isolates from piglets over the five sampling moments.
